# A Short Term Follow Up Comparison of Genu Varum Corrective Surgery Using Open and Closed Wedge High Tibial Osteotomy

**DOI:** 10.5704/MOJ.1303.009

**Published:** 2013-03

**Authors:** Ali Tabrizi, Jafar soleimanpour, Ali Sadighi, Ali Jafari Zare

**Affiliations:** Department of Orthopaedics, Tabriz University of Medical Science, Tabriz, Iran; Department of Orthopaedics, Tabriz University of Medical Science, Tabriz, Iran; Department of Orthopaedics, Tabriz University of Medical Science, Tabriz, Iran; Department of Orthopaedics, Tabriz University of Medical Science, Tabriz, Iran

## Abstract

Introduction: Knee deformity associated with osteoarthritis (OA) is one of the most common complications seen in patients referred to orthopaedic surgeons. High tibial osteotomy (HTO) is an accepted method for treatment of medial knee osteoarthritis with varus deformity. The aim of this study was to compare results of osteotomy methods in patients with genu varum (GV) deformity. Methods: In this cohort study, the sample consisted of 32 patients with genu varum deformity (42 knees) who were divided into two groups and matched according to age and gender. The patients were treated with open or closed wedge osteotomy. After surgery, they were followed-up and compared for 6 months. Results: The sample consisted of 25 women (87.2%) and 7 men (21.8%). Ten patients (31.2%) presented with bilateral deformity. The incidence of complications was the same for both procedures (12.5%); this included one peroneal nerve injury following closed wedge surgery. Overall, patient satisfaction was 87.5% and 75% for the open and closed wedge methods respectively. Operative time, days to full weight bearing, and days to return to routine activities were significantly shorter for patients treated with the open wedge method (p<0.001). Conclusion: Open wedged HTO is associated with shorter operating time, shorter recovery time, higher patient satisfaction, and probable lower risk of neurological injury.

**Key Words:**

High Tibial Osteotomy, Open Wedge Osteotomy, Closed Wedge Osteotomy

## Introduction

Genu varum (GV) is a knee deformity in which the centre of the knee joint is located lateral to the lower limb mechanical axis. Underlying causes for development of this deformity included vitamin D deficiency during childhood, congenital factors, calcium and phosphorus metabolism diseases, trauma and infection1. GV is not desirable due to its awkward appearance and aesthetic issues. Further, it may lead to walking problems and medial knee pain due to inappropriate patella movement in severe cases. In most cases, the deformity gradually destroys cartilage of the knee joint, resulting from unequal pressure imposed on the medial and distal compartments of the knee 1.

High tibial osteotomy (HTO) is widely used to treat osteoarthritis and GV deformity of the knee^2^. Surgical techniques for HTO include open wedge, closed wedge, and dome shaped osteotomy, with open and closed wedge osteotomy being the most common treatment method^3^. Closed wedge osteotomy (CWO), introduced by Coventry in 1969, is performed proximal to the tibial tubercle and a wedge from lateral side is removed^6^. Correction of the GV deformity leads to decreased force on the medial compartment, and improved knee movement and function^4^-^6^.

Although HTO is generally regarded as an effective treatment, there is uncertainty considering the best choice with this method. Controversy is ongoing about the choice of method to use (open wedge vs. closed wedge), whether bone graft for the open wedge technique, methods of fixation, and whether HTO choice affects results of subsequent arthroplasty^7^. We conducted this study to compare results of open and closed wedge HTO for correction of GV deformity.

## Materials and Methods

This cohort study was conducted at a single hospital on two groups of patients with GV deformity. Written informed consent was obtained from all study participants. Active patients with media exclusion criteria consisted of: symptomatic osteoarthritis of the patellofemoral joint, lateral compartment osteoarthritis of the knee, rheumatoid arthritis, history of fractures or history of previous open surgery of lower limbs, or flexion contracture.

Thirty-two GV patients were divided into two equal groups and underwent open or closed high tibial osteotomy. We performed closed wedge osteotomy in patients with intact medial and lateral collateral ligaments, and chose open wedge osteotomy for patients with laxity of the medial collateral ligament. The two groups were matched for age, gender, and BMI.

The degree of knee pain at standing and activity, pre- and postoperatively was evaluated using a visual analogue score (VAS) score. The Lysholm and Wallgren-Tenger Activity Questionnaire score, BMI, plain radiography taken in standing position (both knees), anterior and posterior slope, patella height (based on the Insall Salvati index), stability of lateral and medial collateral ligaments, and knee joint range of motion (ROM) were determined before and after surgery. All the patients were reevaluated one week and 6 months after surgery. Other measures included days to return to work or routine activities, days to complete weight bearing, patient satisfaction, operative time, as well as complications observed during surgery, immediately postoperative, and during the follow-up period. All surgeries were performed by the same surgeon.

For the open wedge method, a skin incision was made over the medial aspect of the proximal fibula, and a second incision was over the medial aspect of the tibia tubercle. The osteotomy was approximately 3.5-cm distal to the articular surface and directed towards the tip of the proximal fibula. Bone ends were separated from the medial side. We used either autograft from the pelvis or allograft, and fixed the osteotomy using L or T plates.

In the closed wedge method, the skin incision was over the lateral aspect of the proximal tibia and we removed a piece of bone in the form of a wedge from the lateral region. Size of the wedge was calculated using this formula: base of wedge = diameter × 0.02 × angle desired. We then closed the osteotomy closed together and fixed them using a plate.

We analysed study data using descriptive statistics (frequency-per cent, mean ± standard deviation). For quantitative and qualitative data, we used T-test (independent samples, paired samples) and Chi-Square or Fischer exact test, respectively. In all cases, a p value of less than 0.05 was considered significant.

## Results

Study participants included 25 women (78.2%) and 7 men (21.8%), with mean age of 35.8±8.8y (range, 19- 46y). We noted decreased articular space of the medial compartment in 21 cases (65.6%). We compared demographic characteristics and found no significant difference between the two groups (Table I). Ten patients (31.2%) had bilateral GV. and 22 (67.8%) unilateral GV. We used the closed wedge technique on 21 limbs and the open wedge method on 21 limbs. For the 10 patients with bilateral GV, 5 underwent open wedge, and the other 5 closed wedge osteotomy surgery.

Complications at the time of surgery were noted in 2 cases (12.5%) in both groups. The complications observed for open wedge method included two cases of intra-articular fracture during screw fixation. There was one case of in closed wedge surgery, also occurring at the fixation stage. There was no vascular injury or compartment syndrome and no additional complications during the six month follow-up period for either method.

We found significant difference when comparing the two groups for level of satisfaction with the size and appearance of the surgical scar (p=0.009). Six patients (37.5%) treated with closed wedge method were not satisfied with the size and appearance of the scar. All patients in the open wedge group were satisfied with the scar size and appearance. We found that in the group treated with the open wedge method, 2 patients (12.5%) were dissatisfied due to continuing pain and 4 patent (25%) s were dissatisfied with closed wedge method (one patient because of nerve paralysis and 3 patients for continuing pain). Generally, overall satisfaction level with the surgery was 87.5% and 75% in open wedge and closed wedge methods respectively.

Other qualitative variables of the groups are shown in Table II. Duration of surgery in open wedge method was significantly shorter than that of the closed wedge technique. There was no significant difference in posterior slope (angle between tibial shaft and articulate surface in lateral radiography) between the two groups before surgery and 6 months after surgery, although the differences observed before and 6 months after surgery in each group were significant (P<0.001). There was no meaningful difference between the two groups for functional evaluation based on the Lysholm and Wallgren-Tenger Activity Score. According to Insall Salvati Index, no change was observed in patella height before and after surgery in both the open and closed wedge groups. Deformity correction rate before and 6 months after treatment was statistically significant from mean value in varus to mean value in valgus in both groups (P=0.001). There was no difference between the two groups considering pre-treatment varus or valgus value and post surgery values. Differences in time to walking with the aid of crutches with toe touch were significant with an average 30 and 12 days for closed wedge and open wedge patients, respectively (p=0.01). Time to complete weight bearing was 2 months in the open wedge group and significantly later in the closed wedge group. Patients treated with open wedge method returned to their routine activities sooner than those treated with closed wedge method.

## Discussion

Medial knee osteoarthritis is associated with varus deformity meaning that additional force is imposed on the medial compartment. The goal of proximal tibial osteotomy is to change the mechanical axis of the lower extremity and correct abnormal force imposed on the medial compartment^8^. Results of long-term follow-up following HTO show 2-8° of valgus between anatomical axes of the femur and tibia as the normal value. There are also reports of patients who subsequently require joint replacement surgery following open or closed wedge HTO^9^.

Open wedge osteotomy (OWO) has recently attracted attentions due to its lower risk of nerve complications compared to closed wedge osteotomy. As peroneal nerve paralysis following closed wedge HTO is uncommon, this technique has been gaining popularity among orthopaedic surgeons^10^. The theoretical advantage of the open wedge osteotomy method is better anatomical correction in both coronal and sagittal plains 4.

The overall complication rate for HTO varied in different reports. For instance, Wu et al. report a complications rate of5.6%, while Naudie et al. report a rate 34.0%. Other studies describe manifestation complications of tibial osteotomy in 10-20% of osteotomy patient 11,12. In our analysis, the overall complication rate was approximately 12.5%, similar to previous studies, but there was no difference between complication rates when comparing the two methods. As reported in other studies, intra-articular fractures were the most important complication of open wedge osteotomy occurring at fixation stage. The problem was solved during follow-up, and no lasting complications were observed. The prevalence of complications for closed wedge osteotomy was the same as for the open wedge group but we saw two different types of complications (one case of peroneal nerve paralysis in the closed wedge group), but this was not statistically significant. The peroneal nerve paralysis persisted during follow-up period (6 months after surgical treatment). There was one case of intra-articular fracture in the closed wedge group. In the present study, there were no cases of delayed or nonunion. Previously reported rates of infection range from 0.8 to 10.4% for HTOs 6,13,14, while in the current study, there was no observed infection. The recovery rate was the same.

Aglietti et al.^15^ describe excellent treatment results after at least 10 years of follow-up in 64% of patients treated with closed wedge osteotomy, and Rudan^16^ reported perfect treatment results in 70% of cases. In the present study, 87.5% and 75% of patients treated with open and closed wedge method respectively expressed satisfaction with treatment results. Less satisfactory results when using the closed wedge method can be attributed to the single case of peroneal nerve paralysis in our sample. Generally, there was no significant difference in clinical outcomes over the six- month follow-up period. Hoell et al. patients and followed 108 patients for 22 months and proved efficacy of both treatment methods, but they concluded that open wedge osteotomy is better for patients requiring more extensive reconstruction of the medial collateral ligament^17^. Lee et al. reported that the open wedge technique was more advantageous than the closed wedge method because biplanar correction and medial fixation using two metal plates are both simpler with this method^14^. Further, the less invasive nature of the open wedge method compares favourably to the closed wedge method, especially for elderly patients 14.

In the present study, operative time was significantly lower for the open wedge technique, representing another advantage of this method and making it possible to use even in patients with cardiovascular problems and individuals who otherwise cannot undergo long surgeries. Earlier return to routine activities, earlier weight bearing and earlier walking are additional advantages of the open wedge method. The residual scar was more satisfactory with the open wedge method as well.

Tibial slope is an important parameter affecting knee biomechanics. The proximal tibial medial articular surface is inclined posteriorly while the distal articular cortex is perpendicular to the posterior tibial surface. Open wedge osteotomy may result in increased tibial slope as opposed to the closed wedge method that can result in decrease of tibial slope. Slope change leads to changes of tibiofemoral contact point and as a result, increase of ACL (anterior cruciate ligament) potential to bear more imposed force and decreased knee extension 18,19. According to Dejour et al., increased tibial slope results in greater posterior and interior cruciate ligament tension force^20^. We found that tibial slope in the open wedge method was higher than in closed wedge procedures, but not to a significant level.

There are contradictions in the literature regarding correction levels of the valgus angle. Insall et al.^21^ report that an acceptable postoperative correction range of valgus is 5-14°, and Coventry et al., stated that angle correction up to 5° results in long-term improvement^22^. Advantages or disadvantages of valgus overcorrection have not yet been studied^21^. Increased valgus angulation probably leads to higher levels of imposed force towards the lateral this is not aesthetically acceptable. Decreased valgus correction is considered a risk factor for treatment failure by Coventry et al, and leads to higher fail rates. Knees with good alignment have lower risk for treatment failure^7^. Agneskircher et al. reported that correcting varus deformity to zero results in approximately a 45% increase of force imposed from the body axis to the knee, and that better results are obtained by shifting the force laterally and decreasing pressure medially^23^. Lee et al. found that a correction of up to 6° at the tibiofemoral angle using the open wedge method led to ideal treatment results. Similar to our own results, other studies report tibiofemoral angle correction rates of 5-8° and excellent treatment results during six-month follow-up.

Wright et al reported that patellar height decreases in all patients treated with proximal tibial osteotomy^24^ due to elevation of the articular surface in reference to the tibial tubercle in open wedge osteotomy. Noyes et al suggest that there is indeed a decrease in patellar height in 80% of open wedge cases. Insall Salveti index results for the present study show no difference between the two treatment groups regarding changes in patellar height after treatment. This is true in other studies, too.

**Table I:**
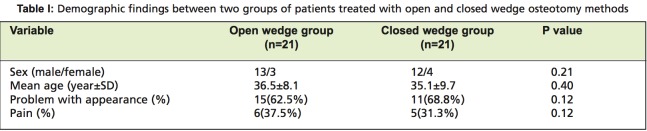
: Demographic findings between two groups of patients treated with open and closed wedge osteotomy methods

**Table II:**
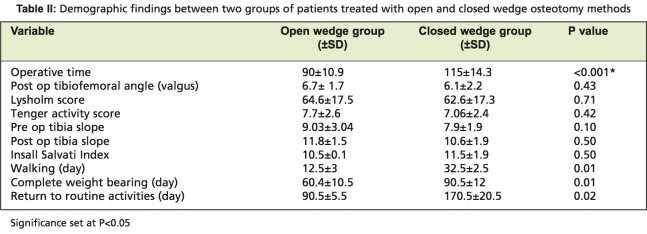
: Demographic findings between two groups of patients treated with open and closed wedge osteotomy methods

## Conclusion

HTO with open wedge osteotomy is more advantageous than the closed technique due to shorter operative time, shorter recovery time, and higher patient satisfaction. There is no difference between the two methods of osteotomy for overall complication rates and surgical outcome, although the risk of neurological injury is probably lower with the open wedge method.
